# Functional fitness normative values for elderly polish population

**DOI:** 10.1186/s12877-020-01787-2

**Published:** 2020-10-06

**Authors:** Zofia Ignasiak, Anna Sebastjan, Teresa Sławińska, Anna Skrzek, Wojciech Czarny, Paweł Król, Marian Rzepko, Barbara Duda-Biernacka, Anna Marchewka, Katarzyna Filar-Mierzwa, Sylwia Nowacka-Dobosz, Janusz Dobosz, Danuta Umiastowska

**Affiliations:** 1 University School of Physical Education in Wrocław, Al. I.J. Paderewskiego 35, 51-612 Wrocław, Poland; 2 University of Rzeszów, Rzeszów, Poland; 3 Gdansk University of Physical Education and Sport, Gdańsk, Poland; 4 University of Physical Education in Krakow, Kraków, Poland; 5 Józef Piłsudski University of Physical Education in Warsaw, Warsaw, Poland; 6 University of Szczecin, Szczecin, Poland

**Keywords:** Elderly, Physical fitness, Norms

## Abstract

**Background:**

It’s really important to measure the actual functional physical fitness of elderly independent living persons from different environments to know the level and rate of decrease may inform about the threat of loss of functional independence, hence the need to monitor and assess the senior’s motor realm and adapt to it the appropriate programs and treatments in the care for the elderly person.

**Methods:**

The study involved 5367 people (mean age 69,63 ± 7,06), including 4164 women (mean age 69,55 ± 7,11) and 1203 men (mean age 69,91 ± 6,86) aged 60 to 93 years old. We have measured basic anthropometric features and physical fitness (by using Senior Fitness Test).

**Results:**

The average values in individual SFT tests significantly decrease along with age. After age of 80 and 85 there were no sex differences in SFT. The largest deficits concern the dynamic balance and the decrease reaches 69% in men and 62% in women A significantly higher rate of decline in aerobic capacity concerns men (43%) than women (36.9%). A clearly lower rate of loss occurs in the muscular strength of the lower and upper body and does not exceed 30%.

**Conclusions:**

The results are of great clinical importance for the development of effective prevention and gerontological education programs in terms of promoting active lifestyle and successful ageing, at the same time limiting the economic consequences of treatment and hospitalisation.

## Background

Dynamic and deep social and economic transformations that began in Poland and other Central European countries, related to the political transformation and the increase in the level of education, translate into clear changes in the lifestyle of people of different ages. This resulted, among others, in a more active approach to one’s own life and health, also in the group of Polish seniors. Hence, the problem of public health is monitoring the level of functional fitness (FF) of the adult and elderly population of Poles, which is an important health factor. Functional Fitness is having the physiologic capacity to perform normal everyday activities safely and independently without undue fatigue [1]. Lifestyle takes the main part in maintaining health and a good quality of life, with physical activity and nutrition being a priority. Many studies prove that the lack of physical activity, alongside the lifestyle (non-communicable) diseases, leads to a decrease in the parameters of physiological ageing, limitation of daily mobility and functional capability [2, 3].

The need to conduct research and assess the physical condition of seniors results not only from dynamic socio-economic changes, but also from very different living, cultural, ethnic, genetic and geographical conditions [4]. The Polish population, as demonstrated by the results of the research by Gronkiewicz [5] and Bielicki et al. [6], is highly genetically and ethnically homogeneous. At the same time, the majority of research conducted among the elderly relates to medical problems and the role of physical activity in the prevention and therapy of many diseases. In parallel to this research, it is extremely important to track the processes of biological ageing among subjectively healthy people living independently. To this, necessary are standardised research tools assessing FF and mapped onto daily activities, such as dressing, preparing meals, hygiene activities, shopping and other. Rikli and Jones [1, 7, 8] have developed and improved a Senior Fitness Test – SFT, that meet safety conditions, are simple and easy to perform, their reliability and accuracy range between 0.79 and 0.97, and the repeatability is in the range of 0.80–0.97.

A big plus in the research of actual FF is the ability to estimate and track the rate of its decrease with age in individual tests [7]. Lowering of the values in physical fitness tests along with age is the expected change, but the level and rate of this decrease may inform about the threat of loss of functional independence, hence the need to monitor and assess the senior’s motor realm and adapt to it the appropriate programs and treatments in the care for the elderly person. It is justified to study the full picture of physical fitness, which has a multidimensional character, and not just individual features, because they do not inform about the general functional state of the senior and the quality of life of the senior [9]. This is very important in creating programs aimed at optimising the biological condition of elderly people.

## Aim of the paper

The main aim of our paper was to provide normative values of FF in Polish seniors. Research results will enable preparing an effective intervention strategy for maintaining mobility and functional independence of elderly people. The development of percentile grids for FF will give the possibility to track the person individually against the background of the population and predict his physical fitness in subsequent years of life.

## Methods

### Study subjects

Our research was cross-sectional and covered a representative of the population sample of people aged 60+ living in Poland – three regions of southern Poland, two regions of northern Poland and one region of central Poland. The study involved 5367 people, including 4164 women and 1203 men aged 60 to 93 years old. Study participants were obtained through senior centres, public institutions and media advertisements. Criteria for inclusion:

- 60 years and above,
- people living independently (not community dwelling, hospitals, nurse homes etc),
- moving and performing daily activities independently,
- no medical contraindications to participate in physical fitness tests,
- no problems in terms of verbal communication,
- no physical and cognitive restrictions.

All participants, if they agreed to take part in the study, prior to the beginning of the research, he signed a voluntary consent form. The research was approved by the Senate Committee for Ethics of scientific research of the University of Physical Education in Wrocław (2015) and were conducted in accordance with the recommendations of the Helsinki Declaration.

The examination of one person took about 50 min and was carried out on 1 day. Measurements and tests of FF were performed in multi-functional or sports halls, in a circuit stations system, with the 6-Minute Walk Test being carried out at the end of the tests [8]. Research was conducted by university staff from Wrocław, Kraków, Rzeszów, Warsaw, Gdańsk and Szczecin, after prior, very thorough training conducted at the Wrocław centre. The research was conducted in 6 cities in Poland and was held from march to october.

### Methods and procedures of functional fitness testing

Functional fitness was assessed using the SFT battery, which consisted of six items, designed and validated by Rikli and Jones [1, 8].

30-Second Chair Stand Test – lower body strength assessment. The examined person sits on a chair of an appropriate height (43 cm high). Arms are crossed at the level of the chest. The task is to perform as many correct lifts (rpts) from the chair as possible within 30 s.

Arm Curl Test – upper body strength assessment. The examined person receives a weight on the dominant hand. The weight of the weight is 2.27 kg [5 lb] for women and 3.63 kg [8 lb] for men. The task is to perform as many correct forearm bends (rpts) as possible within 30 s.

Chair Sit and-Reach Test – assessment of the felxibility of the lower body. The examined person sits down on the edge of the chair. One leg is straightened and the heel rests on the floor (toes pointing to the ceiling). The other leg is bent (the entire foot is on the floor). The task is to bend his hands to the toes as much as possible. The distance from the middle finger to the toes is measured. The result is given with an accuracy of 0.5 cm.

Back Scratch Test – assessment of the flexibility of the upper body. The person is standing and puts one hand on back from top to bottom, the other from bottom to top. The examined person tries to join the fingers of both hands together. The distance between the middle fingers of both hands is measured. The result is given with an accuracy of 0.5 cm.

8-Foot Up-and-Go Test – agility and dynamic balance assessment. The person is sitting on the chair. There is a bollard in front and 2.44 m away from chair. At the signal, the examined person gets up, approaches the bollard, circles it, returns to the chair and sits down. The task should be completed as fast as possible. The result is the time needed to complete the task.

6 – Minute Walk Test – aerobic endurance rating. The examined person moves for 6 min with a fast walk in a rectangle with full dimensions of 45.72 m. The result is the distance covered by the examined person in the given (6 min) time.

### Anthropometric measurements

Height and weight measurements were made using a SECA measuring device model 764. Body Mass Index (BMI) was calculated on their basis. The following categories were adopted: underweight < 18.5 kg/m^2^; norm 18.5–24.9 kg/m^2^; overweight 25–29.9 kg/m^2^; obesity> 30 kg/m^2^, in accordance with criteria provided by WHO.

### Statistical analysis

For statistical calculations, the Statistica v.14 program was used. After checking the normality of the distributions of the examined anthropometric features and the results of functional fitness tests with the Shaphiro-Wilk method, the paper presents the means (M) and standard deviations (SD) in groups of sex and age. Differences between means in the groups were analysed using two-factor analysis of variance ANOVA with NIR post hoc test. The F statistic was counted for main effects as well as sex and age interaction effects, and the significance of inter-group differences was assessed. The significance level of *p* ≤ 0.05 was adopted in all analyses. The rate of decline in the functional fitness of women and men was assessed in percentage values, assuming the average value in the age group of 60–64 to be 100%. In order to quickly and easily diagnose functional fitness of the elderly, percentile grids were constructed. The 5th, 10th, 25th, 50th, 75th, 90th and 95th percentile of examined features was calculated for each 5-year age and sex group starting from the age of 60 (60–64, 65–69, 70–74, 75–79, 80–84 and above 85 years old). The percentile curves were smoothed with a second-degree polynomial. All calculations were performed using Statistica 10.0 software.

## Results

The general statistical characteristic is included in Table 1.

**Table 1:** Average values of anthropometric features and results of FF tests in age and sex groups

	Men						sex	Women					
Variable	60–64	65–69	70–74	75–79	80–84	85+	age [years]	60–64	65–69	70–74	75–79	80–84	85+
Height [cm]	174.2 6.5	172.9 6.0	171.7 6.1	169.0 6.4	168.0 5.7	164.1 6.1	M SD	160.2 5.9	159.0 5.9	157.8 6.0	157.3 6.0	156.3 6.1	154.9 7.9
Weight [kg]	86.7 12.9	87.5 12.8	85.4 12.4	79.0 10.9	77.3 9.4	80.2 12.8	M SD	73.1 13.4	72.1 12.6	70.8 11.7	70.5 11.4	68.9 11.0	67.8 12.4
BMI [kg/m^2^]	28.5 3.8	29.2 3.7	28.9 3.9	27.6 3.4	27.4 2.9	29.7 3.8	M SD	28.5 5.2	28.5 4.8	28.4 4.4	28.5 4.4	28.2 4.5	28.3 5.0
30-Second Chair Stand Test [rpts]	16.1 5.4	16.6 5.3	16.1 5.1	15.3 5.0	14.1 4.8	12.0 5.0	M SD	15.7 4.3	15.1 4.3	14.6 4.2	13.4 4.3	13.0 3.4	11.4 3.4
30-Second Arm Curl Test [rpts]	20.8 5.6	20.7 5.2	18.8 5.2	17.8 5.0	16.2 4.8	14.6 4.6	M SD	18.8 5.2	18.2 5.1	17.6 4.9	16.0 4.6	15.3 3.9	12.9 4.2
Chair Sit-and-Reach Test [cm]	1.8 10.2	0.6 10.9	−0.8 11.2	0.5 10.5	−1.4 11.6	- 4.1 11.1	M SD	4.8 9.4	4.1 9.1	2.9 8.6	2.2 8.9	2.1 9.4	−2.3 12.9
Back Scratch Test [cm]	−8.3 13.3	−9.5 12.2	−10.4 12.8	−12.6 12.3	− 13.9 13.7	− 21.3 10.8	M SD	− 2.4 9.0	−3.5 9.6	−4.1 9.8	−6.3 11.5	−6.4 11.0	− 12.3 12.6
8-Foot Up-and-Go Test [s]	5.8 2.2	5.9 2.1	6.1 1.8	6.8 2.8	7.5 2.5	9.8 3.5	M SD	6.1 1.9	6.4 2.1	6.7 1.9	7.6 2.4	7.6 2.0	9.9 3.7
6-Minute Walk Test [m]	565.1 113.8	549.6 119.5	524.2 140.4	461.4 123.7	440.7 127.4	322.1 118.8	M SD	497.9 109.8	470.7 104.8	440.0 102.9	411.9 105.0	366.8 108.1	314.0 140.0

The basic anthropometric features are differentiated by both the age and sex of the subjects (Table 2). Men were significantly taller and heavier compared to the groups of their peer women. The BMI index values do not show dimorphic differences and remain at similar level in all age groups, indicating dominance of overweight.

**Table 2:** The impact of age and sex on the development of anthropometric parameters and results of tests of FF - main effects of two-factor analysis of variance

Variable	sex		Age		interaction sex-age	
	F	*p*	F	*P*	F	*p*
Height	1264.20	0.0000^*^	43.10	0.0000^*^	4.30	0.0006*
Weight	267.92	0.0000^*^	15.29	0.0000^*^	4.75	0.0003^*^
BMI	0.30	0.5850	2.22	0.0499^*^	1.93	0.0856
30-Second Chair Stand Test	33.75	0.0000^*^	34.44	0.0000^*^	2.41	0.0343^*^
30-Second Arm Curl Test	63.44	0.0000^*^	75.41	0.0000^*^	2.06	0.0674
Chair Sit-and-Reach Test	44.15	0.0000^*^	13.58	0.0000^*^	0.92	0.4644
Back Scratch Test	207.49	0.0000^*^	33.39	0.0000^*^	0.63	0.6792
8-Foot Up-and-Go Test	34.48	0.0000^*^	69.32	0.0000^*^	2.15	0.0563
6-Minute Walk Test	87.78	0.0000^*^	85.73	0.0000^*^	2.24	0.0476^*^

*p < 0.05

The average values in individual SFT tests significantly decrease along with age. Their level is higher among men than women except for two flexibility tests in which women achieved a significantly higher level (Tables 1, 2). The main effects indicate the statistical significance of most tests, only in tests assessing the range of upper and lower body movements there were no statistical significance. The results of the NIR test used for male-female comparisons are interesting. After the age of 80, significant sex differences in the strength of the upper and lower body muscles, agility and dynamic balance disappear, and after the age of 85 they also do in aerobic capacity (Table 3).

**Table 3:** NIR post hoc test for male-female comparisons (bold print *p* ≤ 0.05)

Variable	Men – Women					
Age group [years]	60–64	65–69	70–74	75–79	80–84	85+
Height	0.0000^*^	0.0000^*^	0.0000^*^	0.0000^*^	0.0000^*^	0.0000^*^
Weight	0.0000^*^	0.0000^*^	0.0000^*^	0.0000^*^	0.0000^*^	0.0001^*^
BMI	0.9471	0.0528	0.1747	0.1190	0.2561	0.2309
30-Second Chair Stand Test	0.1184	0.0000^*^	0.0000^*^	0.0000^*^	0.0552	0.5022
30-Second Arm Curl Test	0.0000^*^	0.0000^*^	0.0007^*^	0.0001^*^	0.1517	0.2642
Chair Sit-and-Reach Test	0.0000^*^	0.0000^*^	0.0000^*^	0.0490^*^	0.0031^*^	0.3350
Back Scratch Test	0.0000^*^	0.0000^*^	0.0000^*^	0.0000^*^	0.0000^*^	0.0000^*^
8-Foot Up-and-Go Test	0.0399^*^	0.0013^*^	0.0003^*^	0.0000^*^	0.7538	0.7582
6-Minute Walk Test	0.0000^*^	0.0000^*^	0.0000^*^	0.0001^*^	0.0001^*^	0.5517

*p < 0.05

Younger 60-year-old men compared to younger 70-year-olds do not differ only in strength of the lower body and dynamic balance (Table 4). In the remaining decadal intervals, they differ significantly except for the flexibility of the lower body in two of four age groups.

**Table 4:** NIR post hoc test for comparisons of male (M) and female (F) age groups every 10 years (bold print *p* ≤ 0.05)

Variable	Sex	60–64 v. 70–74	65–69 v. 75–79	70–74 v. 80–84	75–79 v. 85+	Sex	60–64 v. 70–74	65–69 v. 75–79	70–74 v. 80–84	75–79 v. 85+
30-Second Chair Stand Test	M	0.9263	0.0020*	0.0003^*^	0.0000^*^	F	0.0000^*^	0.0000^*^	0.0000^*^	0.0000^*^
30-Second Arm Curl Test	M	0.0000^*^	0.0000^*^	0.0000^*^	0.0001^*^	F	0.0000^*^	0.0000^*^	0.0000^*^	0.0000^*^
Chair Sit-and-Reach Test	M	0.0020^*^	0.9380	0.6136	0.0094^*^	F	0.0000^*^	0.0003^*^	0.1917	0.0000^*^
Back Scratch Test	M	0.0177^*^	0.0020^*^	0.0101^*^	0.0000^*^	F	0.0009^*^	0.0000^*^	0.0005^*^	0.0000^*^
8-Foot Up-and-Go Test	M	0.0719	0.0000^*^	0.0000^*^	0.0077^*^	F	0.0000^*^	0.0000^*^	0.0000^*^	0.0000^*^
6-Minute Walk Test	M	0.0003^*^	0.0000^*^	0.0000^*^	0.0000^*^	F	0.0000^*^	0.0000^*^	0.0000^*^	0.0000^*^

*p < 0.05

Among women, the picture of SFT changes is unambiguous and indicates a significant decrease in values in all test samples used (Table 4).

Analysing the rate of decline of individual SFT test parameters along with age in terms of percentages, the largest deficits concern the dynamic balance and the decrease reaches 69% in men and 62% in women (Table 5).

**Table 5:** Percentage changes of physical fitness with age (60–64 years = 100%)

Variable	60–64	65–69	70–74	75–80	80–85	85+
	Men					
30-Second Chair Stand Test [n]	100%	103%	100%	95%	88%	75%
30-Second Arm Curl Test [n]	100%	99%	90%	86%	78%	70%
Chair Sit-and-Reach Test [cm]	100%	82%	62%	85%	53%	13%
Back Scratch Test [cm]	100%	91%	85%	69%	59%	5%
8-Foot Up-and-Go Test [s]	100%	102%	105%	117%	129%	169%
6-Minute Walk Test [m]	100%	97%	93%	82%	78%	57%
	Women					
30-Second Chair Stand Test [n]	100%	96%	93%	85%	83%	73%
30-Second Arm Curl Test [n]	100%	99%	96%	87%	84%	79%
Chair Sit-and-Reach Test [cm]	100%	90%	74%	64%	63%	3%
Back Scratch Test [cm]	100%	89%	83%	61%	60%	2%
8-Foot Up-and-Go Test [s]	100%	105%	110%	125%	125%	162%
6-Minute Walk Test [m]	100%	94%	88%	83%	74%	63%

A significantly higher rate of decline in aerobic capacity concerns men (43%) than women (36.9%). A clearly lower rate of loss occurs in the muscular strength of the lower and upper body and does not exceed 30%.

Tables 6 and 7 show norm values, taking into account the 10th, 25th, 50th, 75th and 90th percentile, in individual SFT tests in relation to each 5-year age group in women (Table 6) and men (Table 7).

**Table 6:** Age-Group (Years) Percentile Norms for Women

	Percentile					
	N	10th	25th	50th	75th	90th
30-Second Chair Stand Test [rpts]						
60–64	1165	10	**13**	**15**	**18**	21
65–69	1189	10	**12**	**15**	**18**	20
70–74	746	10	**12**	**14**	**17**	20
75–79	488	9	**11**	**13**	**16**	19
80–84	379	8	**11**	**13**	**15**	17
≥ 85	127	7	**10**	**12**	**14**	16
30-Second Arm Curl Test [rpts]						
60–64	1163	13	**15**	**18**	**22**	26
65–69	1188	12	**15**	**18**	**21**	25
70–74	745	12	**14**	**17**	**21**	24
75–79	487	10	**13**	**15**	**19**	22
80–84	379	9	**13**	**15**	**17**	20
≥ 85	127	8	**10**	**12**	**14**	16
Chair Sit-and-Reach Test [cm]						
60–64	1166	−5	**0**	**4**	**10**	17
65–69	1191	−6	**0**	**3**	**9**	16
70–74	744	−7	**0**	**2**	**8**	14
75–79	489	−7	**−1**	**2**	**7**	13
80–84	380	−9.5	**0**	**2**	**7**	12
≥ 85	127	−16	**−6**	**0**	**5**	12
Back Scratch Test [cm]						
60–64	1165	−14	**−7**	**0**	**3**	7
65–69	1192	−17	**−9**	**− 1**	**3**	6
70–74	745	−17	**−10**	**−2**	**3**	6
75–79	489	−20	**−13**	**− 4**	**1**	5
80–84	380	−21.5	**−12**	**−4**	**0**	4
≥ 85	127	−25	**−19**	**− 10**	**−4**	−2
8-Foot Up-and-Go Test [s]						
60–64	1166	4.4	**4.9**	**5.7**	**6.6**	8
65–69	1190	4.7	**5.2**	**5.9**	**6.8**	8.2
70–74	746	4.9	**5.6**	**6.3**	**7.3**	8.8
75–79	488	5.3	**6.0**	**7.0**	**8.4**	10.5
80–84	380	5.3	**6.3**	**7.3**	**8.5**	11
≥ 85	127	5.9	**7.3**	**8.2**	**9.5**	11.9
6-Minute Walk Test [m]						
60–64	1162	351	**415**	**500**	**572**	630
65–69	1187	330	**388**	**477**	**542**	602
70–74	742	310	**365**	**446**	**510**	567
75–79	488	284	**348**	**405**	**480**	556
80–84	377	215	**303**	**376**	**430**	502
≥ 85	127	150	**201**	**300**	**392**	480

**Table 7:** Age-Group (Years) Percentile Norms for Men

	Percentile					
	N	10th	25th	50th	75th	90th
30-Second Chair Stand Test [rpts]						
60–64	291	10	**13**	**16**	**19**	23
65–69	320	10	**13**	**16**	**19**	23
70–74	260	10	**12**	**15**	**19**	23
75–79	176	10	**12**	**14**	**17**	22
80–84	82	9	**11**	**13**	**16**	20
≥ 85	36	7	**9.5**	**11**	**13**	18
30-Second Arm Curl Test [rpts]						
60–64	291	14	**17**	**20**	**25**	28
65–69	320	15	**17**	**20**	**23**	27
70–74	260	13	**16**	**18**	**21**	26
75–79	176	12	**14**	**18**	**20**	24
80–84	82	11	**13**	**16**	**18**	23
≥ 85	36	9	**12**	**14**	**16.5**	20
Chair Sit-and-Reach Test [cm]						
60–64	291	−13	**−2**	**2**	**8**	14
65–69	320	−14	**−4**	**0.5**	**6**	13
70–74	260	−16.5	**−7.5**	**0**	**7**	12
75–79	176	−14	**−4**	**1**	**7**	13
80–84	82	−18	**−9**	**0**	**6**	13
≥ 85	36	−21	**−13.5**	**−1.5**	**3.5**	11
Back Scratch Test [cm]						
60–64	290	−25	**−17**	**−8**	**2**	6.5
65–69	319	−25	**−18**	**− 10**	**0**	5
70–74	260	−27	**−20**	**− 10**	**−2**	5,3
75–79	176	−29.3	**−21**	**−13**	**−4.5**	2
80–84	82	−30	**−22**	**−14.5**	**−6**	4
≥ 85	36	−35	**−26**	**− 20**	**−13.5**	−9
8-Foot Up-and-Go Test [s]						
60–64	291	4.2	**4.6**	**5.3**	**6.2**	8
65–69	320	4.3	**4.8**	**5.5**	**6.5**	8.8
70–74	260	4.4	**5.0**	**5.8**	**6.7**	9.1
75–79	176	4.6	**5.1**	**6.0**	**7.3**	9.7
80–84	82	4.8	**5.8**	**7.0**	**8.5**	10.6
≥ 85	36	4.9	**5.9**	**8.2**	**9.9**	12.0
6-Minute Walk Test [m]						
60–64	290	420	**508**	**572**	**630**	706
65–69	317	381	**475**	**559**	**628**	695
70–74	258	333	**445**	**540**	**625**	694
75–79	176	280	**380**	**475**	**544**	615
80–84	82	298	**362**	**450**	**512**	585
≥ 85	36	156	**215**	**304**	**395**	478

## Discussion

The challenge of modern gerontology is to extend the average life that is free from disability and improve the quality of life in old age. An extremely important element of successful ageing and senility is the ability to function independently. Although the loss of independence cannot be treated as a consequence of physiological ageing, this phenomenon escalates along with age. These decreases in functional mobility cause a threat or a loss of functional independence in everyday life [10, 11].

Obtained results of own research show a picture of FF of Polish seniors, confirming its decline along with age. The presented test values deviate slightly from the norms stated in the papers of Rikli, Jones [8]. It seems that due values shown in own research for each age group are more adequate for the studied group of Polish seniors, i.e. covering a representative sample of people aged 60+ living in Poland. The study involved 5367 people, aged 60 to 93. The studies of Gronkiewicz [5] and Bielicki et al. [6] show that the population of post-war Poland has become very homogeneous in the sense of decrease in the regional and social differences. Thus, in the present population of elderly people, the level of biological condition and possible differences are rather related to the level of social and economic development, living standards and lifestyle. Whereas the norms given by Rikli, Jones [8] concern the multinational American population. Therefore, it seems important to systematically test people and create a database to develop FF standards of Polish seniors from different communities (community dwelling and non-community dwelling as well).

Physical fitness is definitely one of the most important factors of positive health and well-being. It is the main element of health promotion activity, reducing the risk of developing lifestyle diseases. The deterioration of physical fitness is primarily facilitated by changes taking place in the musculoskeletal system and in the system of posture and balance control, conducive to falls and injuries. The loss of physical fitness of the elderly is a problem not only for the patient himself, but is also a social problem, due to the increase in costs related to treatment and care.

Many studies indicate relationships between physical activity and physical fitness, and health [12, 13]. As we know, optimal physical activity of seniors not only prevents many lifestyle (non-communicable) diseases but also extends human life [14–16]. A passive in terms of movement lifestyle increases the risk of illness and disability, especially in seniors. That is why one of the main public health problems is the care for the optimal biological condition of seniors and constant monitoring of their health and lifestyle. Systematic physical activity occupies a particularly important place in striving to slow down adverse biological and non-biological changes associated with ageing. The beneficial result of physical exercise of elderly people is unequivocal, and their effect does not weaken with age [17]. Scientific research shows that increased motor activity has a comprehensive effect on the whole body [15], and especially on the locomotor system [16, 18, 19], as well as circulation and breathing [2, 17], nervous one [16], and reduces the risk of falls [20–22]. Some changes in the human body attributed to the ageing process itself are actually caused by the deficiency or lack of physical activity as well. Doing physical exercises can contribute to the reduction of these changes. The development of optimal and at the same time simple algorithms for rehabilitation procedure, aimed at improving independence, safety of locomotion and broadly understood quality of life, has become a challenge for health sciences [20, 23].

In recent years, the significant promotion of an active lifestyle has increased the awareness and importance of physical activity for maintaining the health of seniors [11, 14]. It should be emphasised that the studies conducted on physical activity indicate its large differences in European Union countries [24–26]. An analysis of world literature indicates that it is difficult to compare the percentage of people engaging in health-promoting physical activity, due to the multitude of assessment methods and recommendations [27]. Sun and Norman [28], in a meta-analysis conducted in 2001–2011 regarding the implementation of physical activity recommendations by people above 60, demonstrated that the implementation of these recommendations varied significantly, in the range from 2.4 to 83%.

The literature describes many tools allowing for assessment of the functional fitness of an elderly person. This fact proves the unflagging interest of researchers in this issue, but also confirms the presence of certain restrictions in already existing tools [9]. Nevertheless, it is important to try to systematically assess the functional fitness of elderly people, which may allow for the preparation of intervention, treatment, care and rehabilitation programs, as well as assessing the individual rate of the changes of individual features and comparing the results obtained by individuals of the same age and sex. It seems that particularly the rate of decline in functional mobility requires in-depth analyses, given its multi-faceted context and conditioning [9, 29]. In our opinion, tests on actual physical fitness supplemented with physical activity tests using measurement methods would be valuable information about the lifestyle of seniors.

Our research brings extremely valuable information about the actual level of FF of Polish seniors and can be an objective reference point for research conducted in various centres and countries. At the same time, they will allow for an individual assessment of the functional status of a given person against the background of the population and peers, which will allow to take action to improve their own mobility, independent functioning and the scope of necessary intervention and assistance. Recognising the problem as soon as possible and implementing individually tailored actions can reduce the risk of losing independence and the need for institutional care. We also hope that our results will be used for comparisons of FF in international comparative statistics. They will also broaden the knowledge about the biological condition of the population of Central Europe.

## Conclusions

The nationwide sample of seniors that we studied can be considered as representative of the general population of elderly Poles because these are the first such extensive nationwide tests on actual physical fitness as an indirect measure for the assessment of the state of health of seniors. Therefore, the results can be used to track individual changes in physiological ageing not only in our country, but also can be used for international comparative studies. Considering that our research was not only limited to the sphere of FF, we intend in further studies to present the relationship of this physical fitness with socio-economic factors, a subjective sense of health and quality of life, as well as programs concerning preventive healthcare and gerontological education. In the case of elderly people, a subjective and active approach to their own health and the place of an elderly person in society are of key importance for successful ageing, but also for a significant reduction in the financial expenditure allocated to hospitalisation and treatment of this social group. Further use of our experimental research can explain many important and important cause-and-effect relationships in the context of both cultural and economic differences in other countries of Europe and the world.

### Strengths

1. These are the first such extensive nationwide tests on actual physical fitness as an indirect measure for the assessment of the state of health of seniors (non-community dwelling) through the use of the generally recognised and accepted battery tests by Rikli & Jones.
2. A significantly large number of people tested (5367) will enable developing a reference system for this type of research and will allow elderly people to individually compare their functional state with national and global averages.
3. The results are of great clinical importance for the development of effective prevention and gerontological education programs in terms of promoting active lifestyle and successful ageing, at the same time limiting the economic consequences of treatment and hospitalisation.
4. Obtained results can be very helpful for all people caring for the elderly so that they can warn their patients about the risk of losing independence, at the same time recommending an optimal exercise program.
5. A comprehensive SFT assessment is definitely more valuable than individual measurements that provide limited information for elderly people who participate in educational programs aimed at compensating for losses in the level of fitness or its decrease.
6. The advantage of this research is the use of proven and solid research methods.

### Limitations

It is mainly the cross-sectional nature of the research, and the fact that the examined person voluntarily agreed to participate in the project. However, obtaining the consent of the respondent is a necessity in empirical research, recommended by the Helsinki Declaration.

## Data Availability

Data are available upon request due to ethical restrictions regarding. participant privacy. Requests for the data may be sent to the corresponding author.

## Abbreviations

FF: Functional Fitness
SFT: Senior Fitness Test
BMI: Body Mass Index
M: Means
SD: Standard deviations
